# Transcriptomic Profiling of circRNAs in rat Hippocampus after Deep Hypothermic Circulatory Arrest

**DOI:** 10.7150/ijms.82503

**Published:** 2023-04-01

**Authors:** Tianlong Wang, Weidong Yan, Shengqiang Pei, Mingru Zhang, Qiaoni Zhang, Yuan Teng, Gang Liu, Jian Wang, Shujie Yan, Bingyang Ji

**Affiliations:** 1 Department of Cardiopulmonary Bypass, Fuwai Hospital, National Center for Cardiovascular Disease, State Key Laboratory of Cardiovascular Medicine, Chinese Academy of Medical Sciences & Peking Union Medical College, Beijing 100037, China; 2 State Key Laboratory of Cardiovascular Disease, Beijing Key Laboratory for Molecular Diagnostics of Cardiovascular Diseases, Diagnostic Laboratory Service, Fuwai Hospital, National Center for Cardiovascular Diseases, Chinese Academy of Medical Sciences & Peking Union Medical College, Beijing, China; 3 Department of Anaesthesiology, Beijing Tongren Hospital, Capital Medical University, Beijing, China

**Keywords:** Circular RNAs, Deep hypothermic circulatory arrest, Cerebral injury, Rat, Hippocampus

## Abstract

Neurologic abnormalities occurring after deep hypothermic circulatory arrest (DHCA) remain a significant concern. However, molecular mechanisms leading to DHCA-related cerebral injury are still ill-defined. Circular RNAs (circRNAs) are a class of covalently closed non-coding RNAs and can play important roles in different types of cerebral injury. This study aimed to investigate circRNAs expression profiles in rat hippocampus after DHCA and explore the potential functions of circRNAs in DHCA-related cerebral injury. Hence, the DHCA procedure in rats was established and a transcriptomic profiling of circRNAs in rat hippocampus was done. As a result, a total of 35192 circRNAs were identified. Among them, 339 circRNAs were dysregulated, including 194 down-regulated and 145 up-regulated between DHCA and sham group. Gene Ontology and Kyoto Encyclopedia of Genes and Genomes analyses were performed based on the host genes of all dysregulated circRNAs. Also, 4 circRNAs were validated by RT-qPCR (rno_circ_0028462, rno_circ_0037165, rno_circ_0045161 and rno_circ_0019047). Then a circRNA-microRNA (miRNA) interaction network involving 4 candidate circRNAs was constructed. Furthermore, functional enrichment analysis of the miRNA-targeting mRNAs of every candidate circRNA was conducted to gain insight into each of the 4 circRNAs. Our study provided a better understanding of circRNAs in the mechanisms of DHCA-related cerebral injury and some potential targets for neuroprotection.

## Introduction

Deep hypothermic circulatory arrest (DHCA) is widely used in complex cardiothoracic surgeries, such as aortic dissection and congenital heart diseases 1. Circulatory arrest can offer a bloodless surgical field to surgeons and deep hypothermia can decrease the metabolism and protect organs against global ischemia 2. However, when blood contacts the artificial surface of DHCA circuits, the inflammation will be triggered. During the rewarming and reperfusion period of DHCA, it would induce ischemia/reperfusion (I/R) injury 3. The rate of neurologic complication in adults who underwent aortic surgery with DHCA was 13.9% 4. In one recent meta-analysis, the incidence of clinical and electroencephalogram seizures among pediatric patients with DHCA was 12.9% and 14.9%, respectively 5. Neurologic abnormalities occurring after DHCA remain a significant concern.

In clinical practice, selective antegrade or retrograde cerebral perfusion are the common approaches for neuroprotection, but it is still controversial whether they can improve the nervous system outcomes 6, 7. In basic medical research, although, some neuroprotective agents have been prone to improve DHCA-related cerebral injury in the animal models, the clinical effect and application still need to be evaluated 8, 9. This study aimed to deeply evaluate cerebral injury after DHCA from the perspective of genetic changes.

Circular RNAs (circRNAs) are a class of non-coding RNA characterized by a covalently closed ring structure without 5′-3′ polarity 10. A wide range of studies have demonstrated circRNAs can act as microRNA (miRNA) sponges, RNA-binding protein interacting agents and nuclear transcriptional regulator, thus controlling gene expression at transcriptional, post-transcriptional, and translational levels 11. Currently, several studies have shown that there were differences in the expression of circRNAs in different types of cerebral injury 12-14. Several key circRNAs can regulate the process of cerebral I/R injury through a variety of mechanisms via miRNA-messenger RNA (mRNA) axis 15.

Hence, we need to further explore dysregulated circRNAs in the central nervous system after DHCA. The hippocampus is associated with cognition, learning, and memory. Also, it is the most vulnerable to I/R injury 16. Here, we established the DHCA procedure in rats and analyzed the differentially expressed circRNAs (DECs) in the hippocampus. Furthermore, the Gene Ontology (GO), Kyoto Encyclopedia of Genes and Genomes (KEGG) and circRNA-miRNA network pattern analyses were conducted to identify the potential roles of DECs in DHCA-related cerebral injury. Taken together, our results may bring valuable information for researchers to further explore the functional roles of circRNAs and new targets in DHCA-related cerebral injury.

## Materials and Methods

### Animals

The protocols received institutional review and got approval from the Institutional Animal Care and Use Committee, Fuwai Hospital, Chinese Academy of Medical Sciences (FW-2021-0005). All experimental procedures complied with the Guide for the Care and Use of Laboratory Animals published by the National Institutes of Health. Sprague-Dawley rats were kept under standard laboratory conditions, within free access to food and water (provided by the HFK Bioscience, China). Rats (age, 12-14 weeks; weight, 450-550 g) were randomly allocated into 2 groups: sham group, DHCA group (n = 5, each group).

### Deep hypothermic circulatory arrest

DHCA procedures were established as previously described 8, 9. In DHCA group, rats were anesthetized with 2% sevoflurane and incubated with a 16-G endotracheal tube. Then rats were mechanically ventilated at 80 breaths per minute with 10 mL/kg tidal volume. Mean arterial blood pressure (MAP) was monitored through the left femoral artery. The tail artery and the right external jugular vein were cannulated and connected to a DHCA circuit, which contained a reservoir, a membrane oxygenator, and a heat exchanger. The DHCA circuit was primed with 12 mL of 6% hydroxyethyl starch and 2 mL saline with 150 IU heparin. After heparinization (500 IU/kg), cardiopulmonary bypass was initiated at a flow rate of 160-180 mL/kg/min for 10 min. Blood flow was directed from the jugular vein through silicon tubes to the membrane oxygenator and back to rat via the tail artery. The target deep temperature was 18 °C. After 30 minutes of systemic cooling, DHCA was induced by draining the blood to the reservoir and lasted nearly 45 minutes, which was confirmed by MAP = 0. In the rewarming phase, the temperature increased gradually by rewarming the blood in the DHCA circuits. The rewarming phase lasted more than 60 minutes. After that the rats were subjected to 40 minutes for reperfusion to recovery. Then the DHCA circuit was weaned off within another 20 minutes. Finally, rats were ventilated for 30 minutes without cardiopulmonary bypass support. During the whole procedure, MAP was more than 50 mm Hg. Rats in the sham group were only anesthetized, cannulated, and heparinized. At the end of the experiment, euthanasia was performed by cervical dislocation under deep anesthesia. Hippocampus was harvested in liquid nitrogen and stored at -80 °C immediately for further analysis.

### RNA extraction

RNA was extracted from frozen tissue using Trizol (Invitrogen, Beijing, China) following the manufacturer's protocol. RNA purity was measured by spectrophotometry, with all samples having A260/280 ≥ 1.9. The RNA samples were stored at -80°C for sequencing and reverse transcription-quantitative polymerase chain reaction (RT-qPCR) analyses.

### Library construction and sequencing

Library construction and sequencing was carried out by a professional company (Novogene, Beijing, China). Briefly, ribosomal RNAs and linear RNAs were removed by using Ribo-zero rRNA removal kit (Epicentre, USA) and RNase R (Epicentre, USA) respectively to enrich circRNAs. The enriched circRNAs were randomly fragmented into 250~300 bp by RNA Fragmentation Reagents (Ambion, USA). Subsequently, first-strand cDNA and second-strand cDNA were synthesized on the fragments, following end-repair, A-tailing, ligation of adaptors and PCR amplification by using NEBNext Ultra Directional RNA Library Prep Kit for Illumina (NEB, USA). At last, the PCR products were purified using the AMPure XP beads (Beckman Coulter, USA), and libraries were evaluated in the Agilent 2100 bioanalyzer (Agilent, USA) and quantified using qPCR. After library construction, Illumina PE150 (Illumina, USA) was used for further sequencing.

### Pre‑processing of data and genomic alignment

Raw reads were filtered by discarding reads containing adapter, ploy-N or low-quality bases. Then the clean reads were mapped to the reference genome sequence (ftp://ftp.ensembl.org/pub/release-97/gtf/rattus_norvegicus/) using HISAT2 (v2.0.5) 17, and the standard SAM files were generated.

### CircRNA identification and differential circRNA expression analysis

A total of two tools, find_circ and CIRI were used to identify circRNAs 18, 19. Since the circRNAs could not be mapped to the reference genome directly, find_circ extracted unmapped reads and selected both ends of these reads (default 20 bp) as anchor reads. Each pair of anchor reads was aligned to the reference genome sequence again. If the alignment positions of the two anchors were reversed in the linear direction, then the reads were extended to find the junction position of circRNAs. At last, the sequences on both sides containing GT/AG splicing sites would be considered circRNAs candidates. CIRI analyzed CIGAR scores and scanned paired chiastic clipping signals in SAM files. Then the candidate circRNAs could be identified based on junction reads and GT-AG splicing signals, following systematic filtering steps to eliminate potential false positives. Normalized read counts of the identified circRNAs were reported as transcripts per million (TPM). DESseq2 algorithm was conducted to obtain DECs between sham and DHCA groups. The criteria were defined as |log2 fold-change (log2FC) | ≥ 1.5 and *p* < 0.05.

### GO annotation and KEGG pathway analyses

To detect the potential functions of DECs, we conducted GO and KEGG enrichment analyses on the host genes of all DEGs. DAVID online tool was chosen for generating analysis results 20. GO and KEGG terms with *p* < 0.05 were considered significant enrichment.

### RT-qPCR

Total RNA was isolated from stored RNA samples and was then converted into cDNA using PrimeScript™ RT reagent kit with gDNA Eraser (Catalog. #R323-01, Vazyme, Nanjing, China). RT-qPCR was performed using the SYBR Premix Ex Taq™ II (Catalog. #Q511-02, Vazyme, Nanjing, China) according to the manufacturer's protocol. RT-qPCR conditions included: pre-denaturation (95˚C, 5 min, 1 cycle) and PCR reaction (95˚C, 10 sec, 60˚C, 30 sec, 40 cycles in total) followed by a dissolution curve. β-actin was designed as an internal control. The 2^-∆∆CT^ method was used to calculate the relative quantification of DEGs expression levels. Primer sequences used in this study were listed in **Supplemental Table S1**.

### Agarose gel electrophoresis for the validated circRNAs

Agarose gel electrophoresis procedure was conducted as previously described 21, 22. Firstly, we performed PCR enrichment of the divergent primers of 4 circular RNAs in both cDNA and gDNA by using 1.1 × T3 Super PCR Mix (TsingKe, Nanjing, China). PCR conditions included: pre-denaturation (98˚C, 30 sec, 1 cycle) and PCR reaction (98˚C, 15 sec, 60˚C, 10 sec, 72˚C, 10 sec, 35 cycles in total) followed by 72˚C for 7 min in 1 cycle. Then, the PCR products were checked on 1% agarose gel electrophoresis. The results are presented in **Supplemental Figure S1**.

### Construction of circRNA-associated ceRNA network

Because circRNAs function as miRNA sponges, we further predicted the target miRNAs of the selected circRNAs as well as the miRNA-targeting mRNAs. CircRNA-miRNA interactions were predicted with miRanda (v3.3a), RNAhybrid (v2.1.1) and TargetScan (v8.0). Then, Cytoscape software (v3.9.1) was conducted to construct the whole circRNA-miRNA interaction network. And TargetScan (v8.0) and miRDB (v6.0), were used to identify target mRNAs of miRNAs in the circRNA-miRNA network. Finally, these target genes were subjected to GO and KEGG analyses.

### Statistical analysis

Data processing and statistical analysis were performed using R (v4.2.1; https://www.R-project.org/ (accessed on 10 July 2022)). The experiment and RT-qPCR results were presented as mean ± SEM. Student's t-tests were used for comparisons be-tween the two groups. *P* < 0.05 were considered to be statistically significant.

## Results

### Transcriptomic analysis of circRNAs in rat hippocampus after DHCA

**Figure 1:**
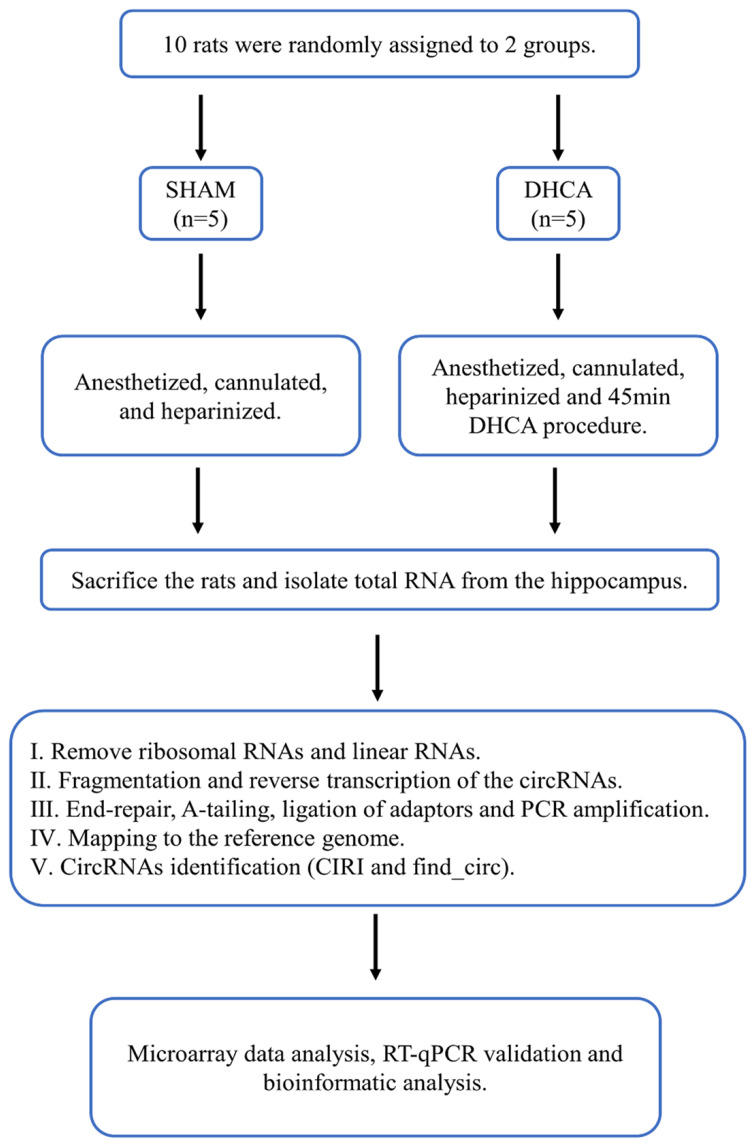
Workflow for the whole study. DHCA, Deep hypothermic circulatory arrest; RT-qPCR, reverse transcription-quantitative polymerase chain reaction.

**Figure 2:**
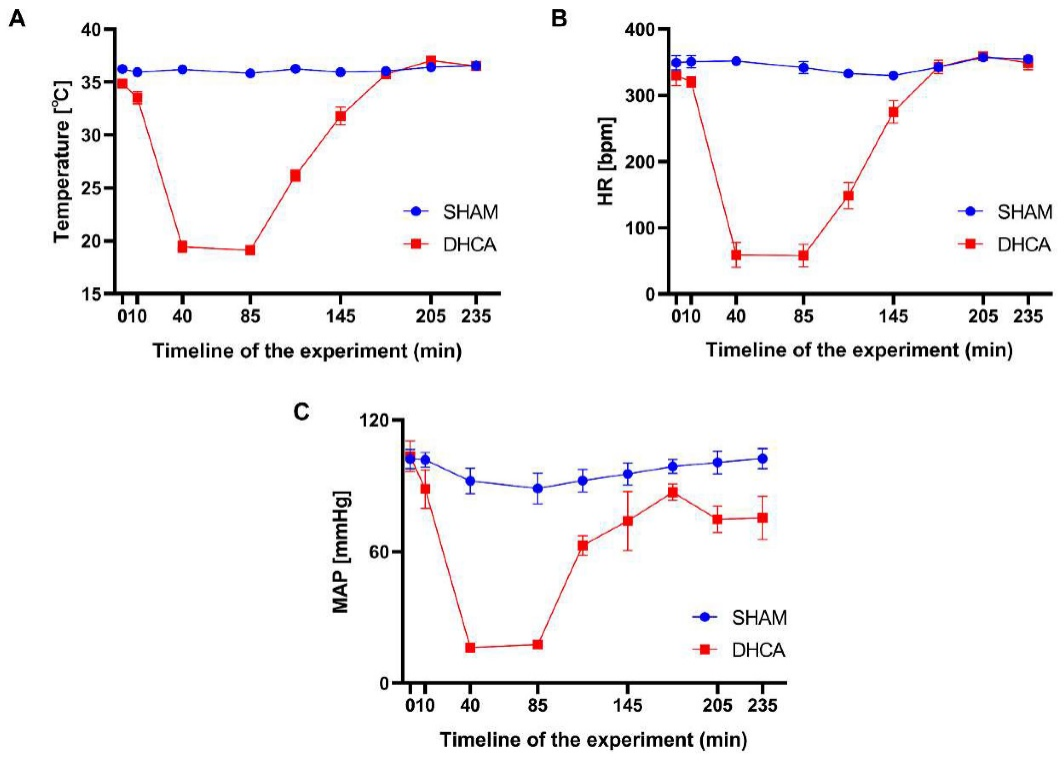
Physiological parameters for the rats throughout the experimental period. Temperature (A), heart rate (HR, B) and mean arterial pressure (MAP, C) were measured over time in the sham and DHCA groups (values: mean ± SEM). bpm, beat per minute. *P* > 0.05 was not shown.

**Figure 3:**
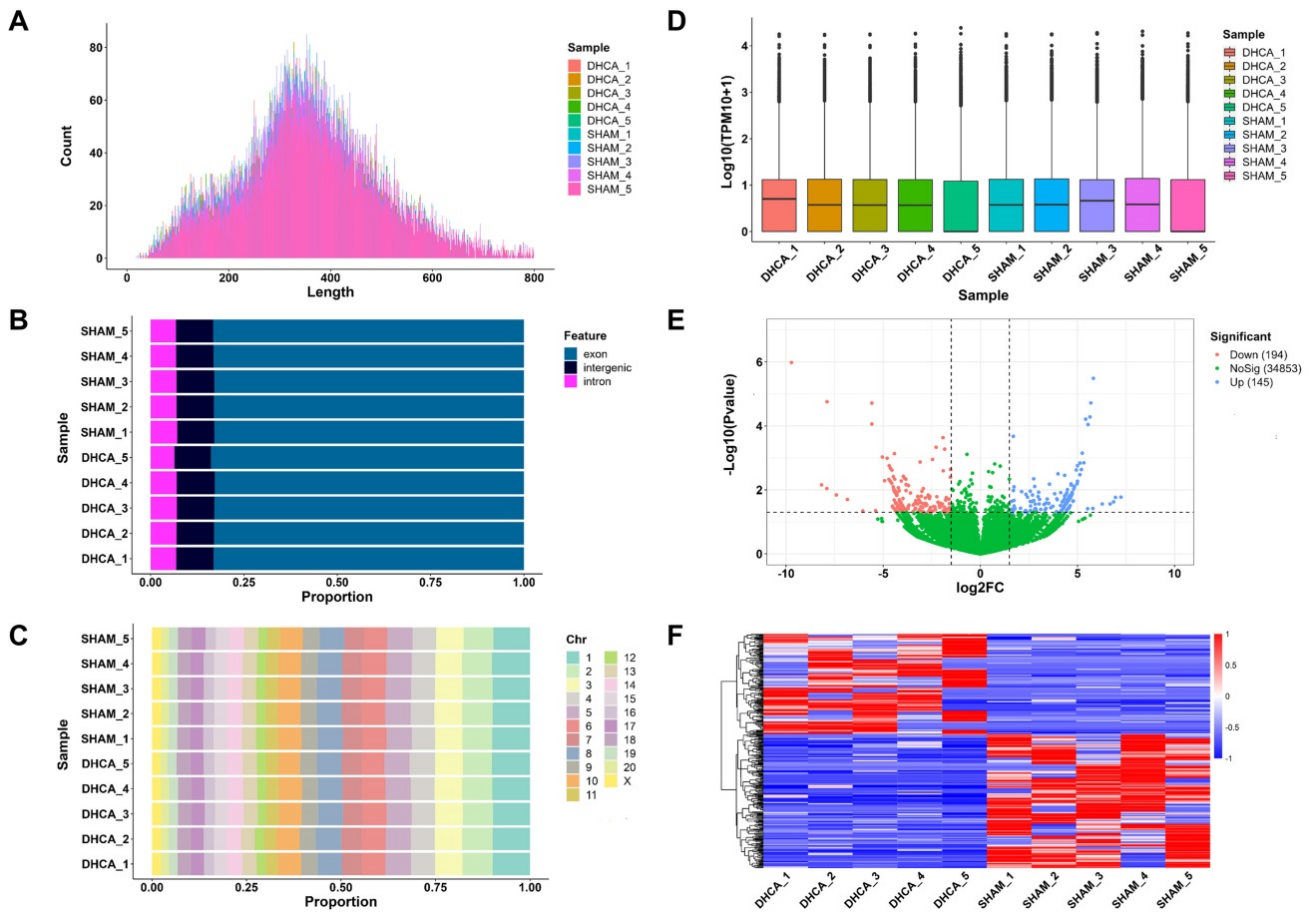
Expression profiling data of circRNAs in the rat hippocampal after DHCA. (A) Length distribution of the identified circRNAs in all samples. (B) Genomic location distribution of the identified circRNAs in all samples. (C) Chromosome distribution of the identified circRNA in all samples. (D) The boxplot of normalized expression values in all samples. (E) Volcano plot analysis for DECs (P < 0.05 and |log2FC| ≥ 1.5 are set as the cut-off criteria). Red, blue, and green points represent circRNAs that are down-regulated, up-regulated, and not significantly different. (F) Heatmap presents the expression of all DECs. Red and blue indicates increased and decreased expression, respectively. DHCA, deep hypothermic circulatory arrest; TPM, transcripts per million; DECs, differentially expressed circRNAs; log2FC, log2 fold-change.

The whole workflow of our study was shown in **Figure 1**. In this study, rats were randomly divided into sham and DHCA groups. The physiologic parameters of the rats throughout the experimental period were illustrated in **Figure 2**. After experiencing 45 minutes DHCA, hippocampus tissue was harvested for circRNAs screening. Finally, a total of 35192 circRNAs were identified from all samples. The length, genomic location, and chromosome distribution of the identified circRNAs were consistent **(Figure 3A-C)**.TPM was used as the final expression value of circRNAs. The TPM distribution of each sample was similar following data pre-treatment** (Figure 3D)**. Then, differential expression analyses were conducted to figure out dysregulated circRNAs between the two groups. Under the criterion as |log2FC| ≥ 1.5 and *p* < 0.05, we found 339 significant DECs, of which 194 were down-regulated and 145 were up-regulated **(Figure 3E)**. A heat map was generated to visualize the differential expression distribution for all DECs **(Figure 3F)**.

### Functional enrichment analysis on the differentially expressed circRNAs

**Figure 4:**
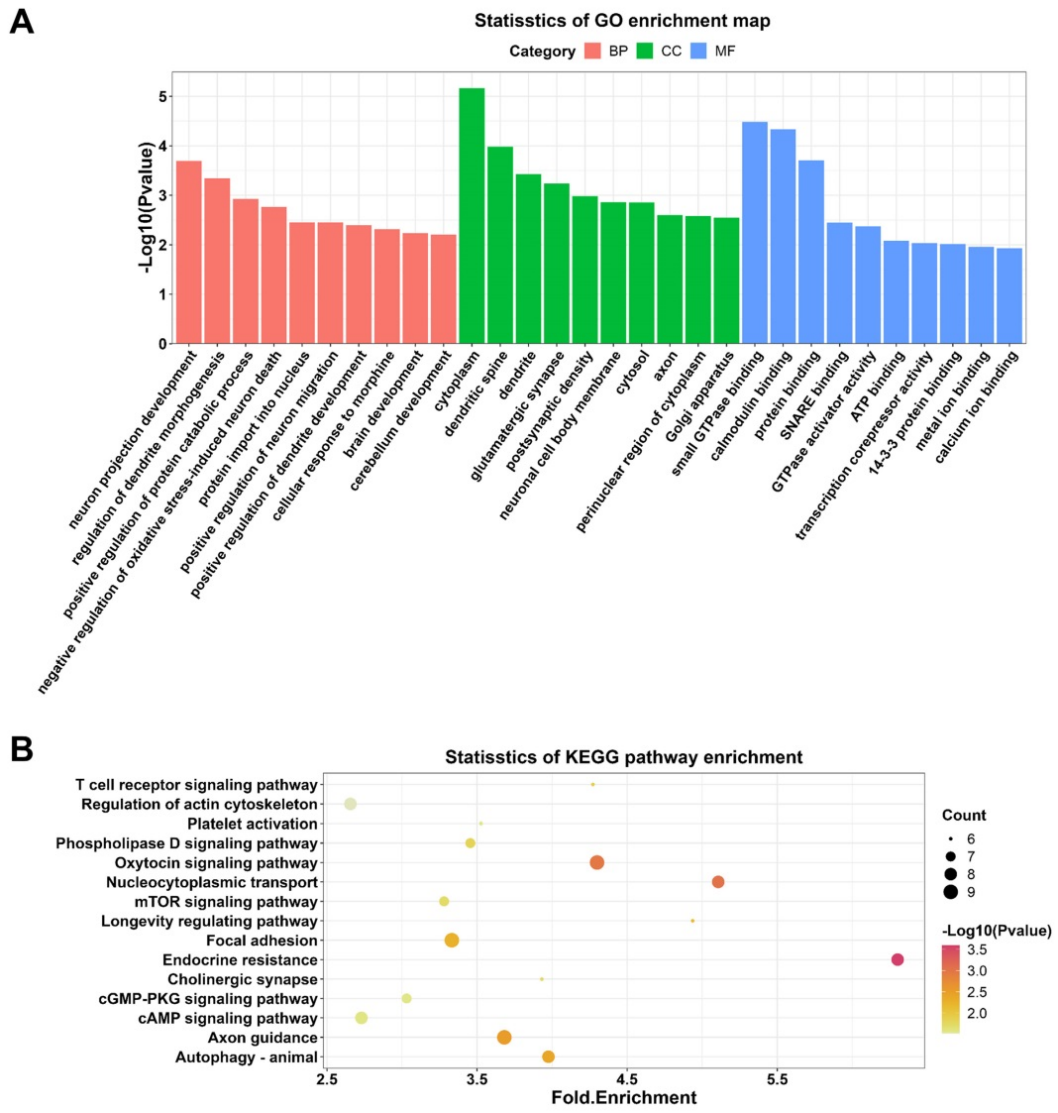
GO and KEGG enrichment analyses on DECs in the hippocampal after DHCA. (A) Top 10 GO terms in the molecular function, cellular component and biological process categories of DECs in the rat hippocampal after DHCA. (B) Top 15 KEGG pathways of DECs in the rat hippocampal after DHCA. GO: Gene Ontology; KEGG: Kyoto Encyclopedia of Genes and Genomes; DECs, differentially expressed circRNAs; DHCA, deep hypothermic circulatory arrest.

To explore the potential functions of DECs, thus GO and KEGG enrichment analyses were conducted. GO analysis enriched 87 terms (*p* < 0.05) within the biological processes, molecular functions, and cellular components, including neuron projection developpment, negative regulation of oxidative stress-induced neuron death, positive regulation of dendrite development, and brain development. These results suggested that these DECs may play an essential role in brain and neuron development. The top 10 GO terms in each category were selected for visualization **(Figure 4A)**. 26 KEGG pathways (*p* < 0.05) were enriched indicating DEGs were associated with T cell receptor signaling pathway, autophagy, cyclic adenosine monophosphate (cAMP) signaling pathway, cyclic guano-sine monophosphate (cGMP)-cGMP-dependent protein kinase (PKG) signaling pathway, and mammalian target of rapamycin (mTOR) signaling pathway. The top 15 KEGG pathways were demonstrated in **Figure 4B**.

### Validation of circRNA by RT-qPCR

**Figure 5:**
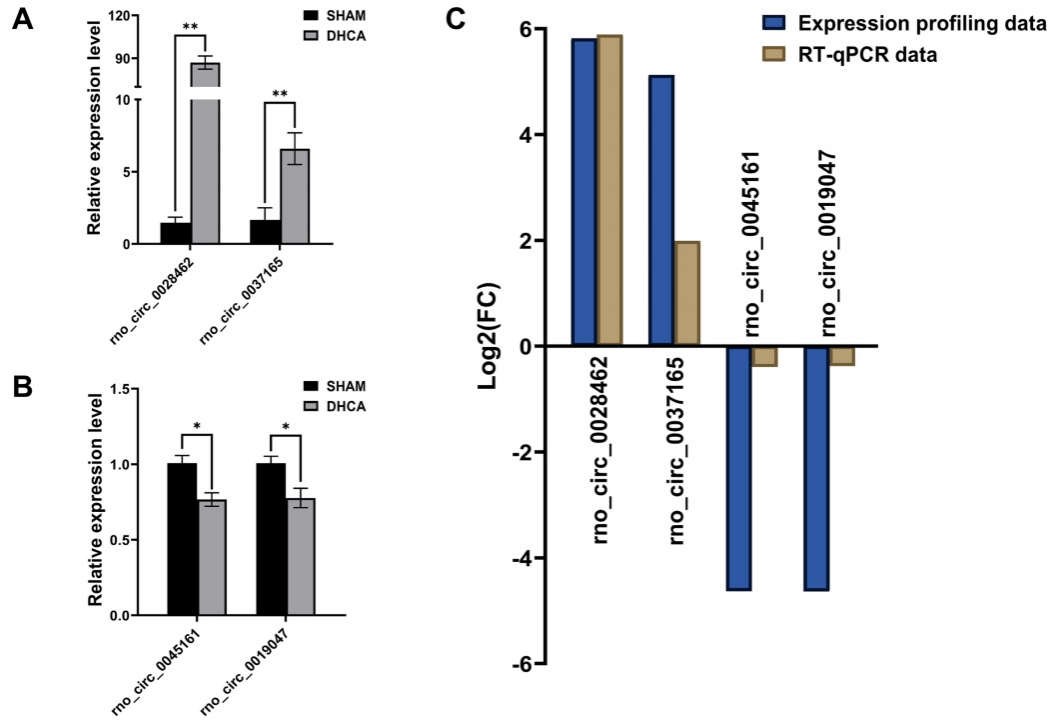
Validation of differentially expressed circRNAs. (A-B) The high expression of rno_circRNA_0028462 and rno_circRNA_0037165 and the low expression of rno_circRNA_0045161 and rno_circRNA_0019047 were confirmed with RT-qPCR method (n = 4, each group). (C) The RT-qPCR data of 4 verified circRNAs displayed the tendency in accordance with our expression profiling results. *p < 0.05, **p < 0.001.

To validate the expression profiling data, we chose 15 circRNAs in the top 30 up-regulated DECs and 15 circRNAs in the top 30 down-regulated DECs for further RT-qPCR validation by using previous hippocampus samples (n = 4, each group). The characterizations of these 30 DECs were listed in **Table 1**. Finally, RT-qPCR analysis revealed 4 DECs were differentially expressed in the DHCA samples compared to the sham group. Rno_circ_0028462 and rno_circ_0037165 were up-regulated, while rno_circ_0045161 and rno_circ_0019047 were down-regulated significantly** (Figure 5A and B)**, which were also in accordance with our expression profiling results **(Figure 5C)**. The genomic location and sequence information of the 4 validated circRNAs were visualized by using Integrated Genome Viewer and presented in **Supplemental Table 2.**

### CircRNA-miRNA interaction network

**Figure 6:**
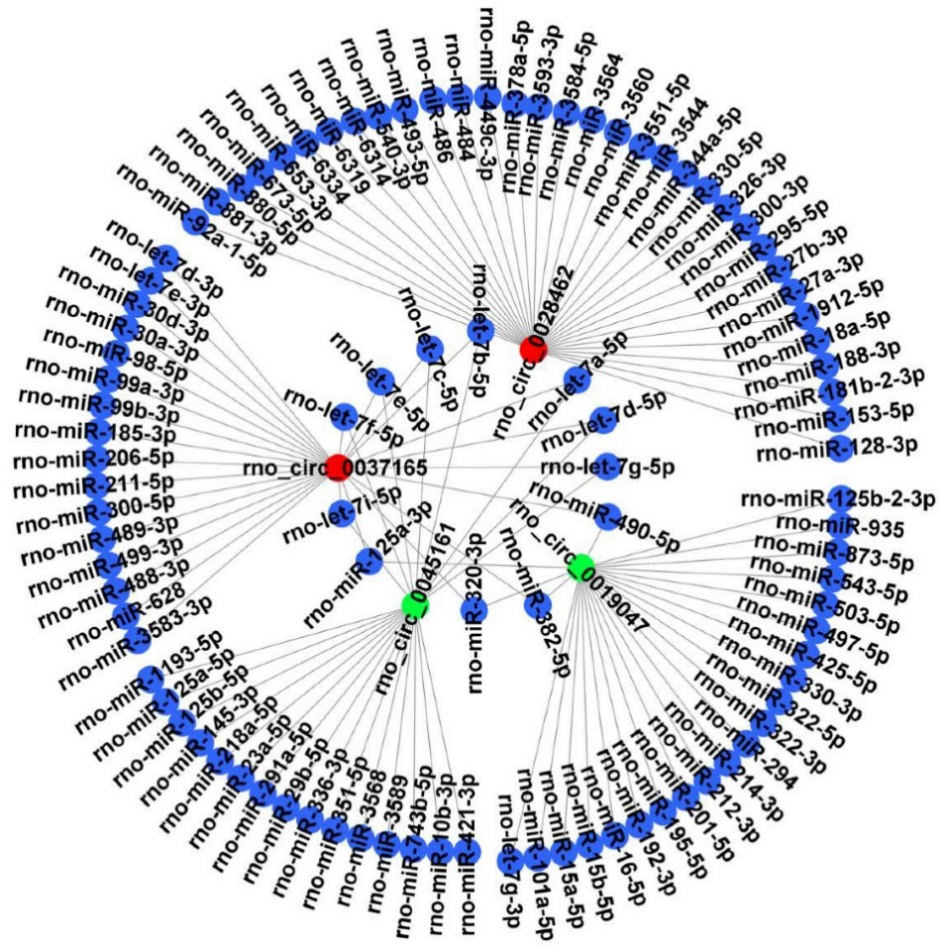
CircRNA-miRNA interaction network. The 4 DECs (rno_circRNA_0028462, rno_circRNA_0037165, rno_circRNA_0045161 and rno_circRNA_0019047) verified by RT‑qPCR were selected to construct a representative circRNA-miRNA network. Red represents up-regulated DECs. Green represents down-regulated DECs. Blue represents the targeted miRNAs. DECs, differentially expressed circRNAs.

It is well known that circRNAs can bind target miRNA and then influence the miRNA-targeted mRNA. Thus, 4 candidate circRNAs verified by RT‑qPCR were selected to construct a representative circRNA-miRNA network **(Figure 6)**.

**Figure 7:**
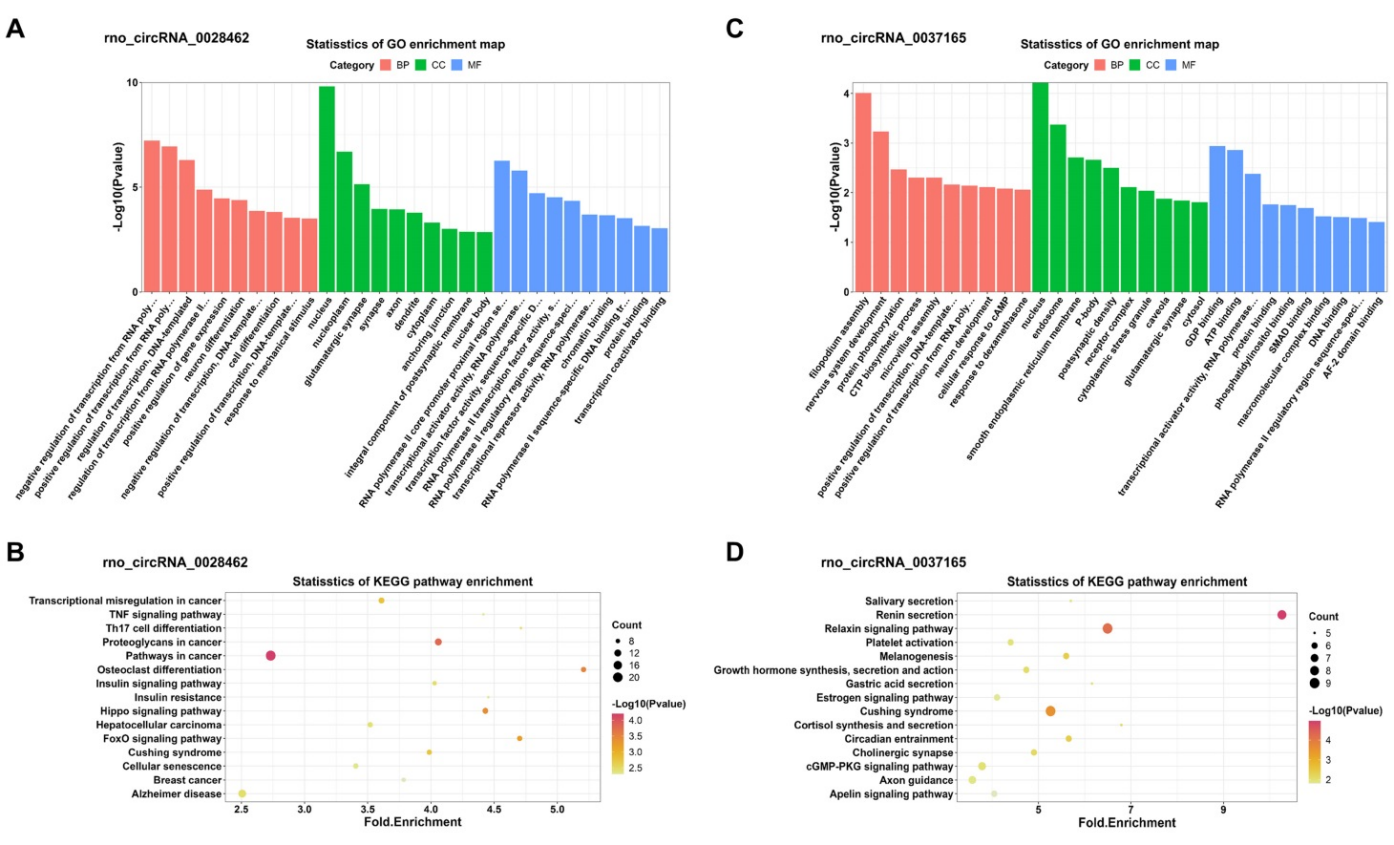
GO and KEGG enrichment analyses of the miRNA-targeted mRNAs of 2 confirmed up-regulated DECs. (A, C) Top 10 GO terms in the molecular function, cellular component and biological process categories of the miRNA-targeted mRNAs of rno_circRNA_0028462 and rno_circRNA_0037165, respectively. (B, D) Top 15 KEGG pathways of the miRNA-targeted mRNAs of rno_circRNA_0028462 and rno_circRNA_0037165, respectively. GO: Gene Ontology; KEGG: Kyoto Encyclopedia of Genes and Genomes; DECs, differentially expressed circRNAs.

**Figure 8:**
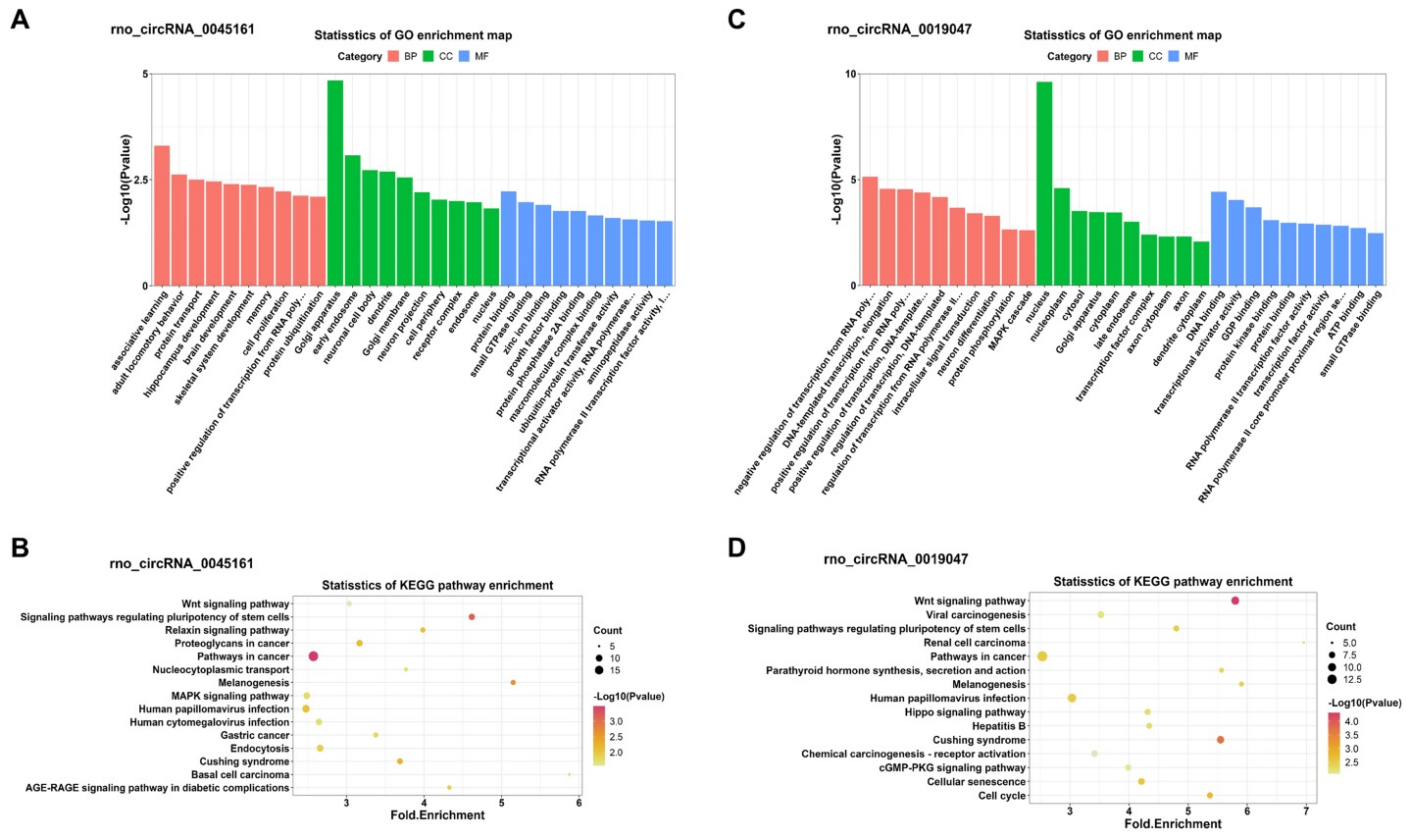
GO and KEGG enrichment analyses of the miRNA-targeted mRNAs of 2 confirmed down-regulated DECs. (A, C) Top 10 GO terms in the molecular function, cellular component and biological process categories of the miRNA-targeted mRNAs of rno_circRNA_0045161 and rno_circRNA_0019047, respectively. (B, D) Top 15 KEGG pathways of the miRNA-targeted mRNAs of rno_circRNA_0045161 and rno_circRNA_0019047, respectively. GO: Gene Ontology; KEGG: Kyoto Encyclopedia of Genes and Genomes; DECs, differentially expressed circRNAs.

In addition, KEGG and GO analyses for miRNA-targeted mRNAs of every DEC were conducted to gain insight into each of the 4 DECs. GO analysis suggested that the miRNA-targeted mRNAs of the 4 DECs were mainly involved in transcription-associated terms. KEGG analysis enriched many key regulatory signaling pathways, like tumor necrosis factor (TNF), forkhead box O (FOXO), Hippo, cGMP-PKG, mitogen-activated protein kinase (MAPK) and wingless / integrated (Wnt) signaling pathways. The top 10 enriched GO terms in the three categories and the top 15 KEGG pathways were shown in **Figure 7** and **Figure 8** respectively.

## Discussion

To provide insights into the understanding of the DHCA pathophysiology, we analyzed the expression profiles of circRNAs in rat hippocampus via a DCHA rat model. A total of 35192 circRNAs were identified by using high-throughput sequencing technology. Among them, 339 circRNAs were dysregulated, including 194 down-regulated and 145 up-regulated between DHCA and sham groups. Furthermore, we verified 4 circRNAs by RT-qPCR (rno_circ_0028462, rno_circ_0037165, rno_circ_0045161 and rno_circ_0019047). Our results will provide new targets for interventions to attenuate cerebral injury induced by DHCA.

Various underlying mechanisms take part in cerebral injury after DHCA, including systemic inflammatory response syndrome, oxidative stress, and blood brain barrier (BBB) damage 23-25. During the circulatory arrest period, the cerebral blood supply is suspended and restored during the reperfusion period, which increases the risk of I/R injury to brain. At present, a large number of studies have shown that circRNAs regulated ischemia-induced brain injury 15, 26. Our results confirmed that the expression profiles of circRNAs in rat hippocampus changed after the DHCA procedure, which was consistent with other studies 27. However, the number of total circRNAs and dysregulated circRNAs in our results was far more than in the previous study 27. Many reasons attribute to this difference, such as the differences in sequencing methods and experimental environments. But we think that one of the reasons was most likely that our sample size in each group (n = 5) is larger than that (n = 3) in the previous study.

Via splicing machinery competition between linear and circular transcripts, circRNAs could interact with their host genes and affect their expression 28. Additionally, some circRNAs remain in the nuclei and regulate the expression of their host genes by interacting with chromatin and/or their promoters 29. Therefore, we examined the potential functions of our DECs by running GO and KEGG enrichment analyses on their host genes. GO results showed that DECs were associated with neuronal development. Prior studies have demonstrated that circRNAs are enriched in the mammalian brain and closely linked to neuronal development such as proliferation and differentiation of neural stem cells 30, 31, thus our result was reasonable. KEGG analysis included various signaling pathways (cAMP, cGMP-PKG, mTOR signaling pathways), which may involve in the regulation of DHCA-related cerebral injury. For example, activation of either the cAMP pathway or the cGMP pathway can phosphorylate the cAMP response element binding protein (CREB) 32. In DHCA, increased phosphorylated CREB may attenuate the severity of brain injury 33. The mTOR can be activated by the phosphatidylinositol 3' -kinase (PI3K)-protein kinase B (PKB/AKT) pathway, decreasing apoptosis and protecting the brain against I/R injury 34. The mTOR also can be regulated by AMP-activated protein kinase (AMPK), resulting in the promoting of autophagy and ameliorating cerebral I/R injury 35. In addition, our results indicated that several DECs were enriched in the autophagy pathway. So far, few studies investigated the autophagy of the central nervous system under DHCA condition. But researchers presented that the role of autophagy in DHCA-mediated lung injury varies with time recently. Autophagy showed protective inhibition at 3 hours after DHCA, but overactivated at 6 hours after DHCA, aggravating lung injury 36. Together with the DECs in the autophagy pathway of the hippocampus, it is necessary to further evaluate the autophagy in cerebral injury induced by DHCA in the near future.

It is widely accepted that circRNAs can act as miRNA sponge or competing endogenous RNA (ceRNA) 11. However, research on the ceRNA function of circRNAs in DHCA is limited. Therefore, we predicted the miRNAs of 4 candidate circRNAs verified by RT-qPCR (rno_circ_0028462, rno_circ_0037165, rno_circ_0045161 and rno_circ_0019047). Wang et al. analyzed the expression of miRNA in piglet hippocampus after DHCA and a number of dysregulated miRNAs were identified, such as miR-10b, miR-23a and miR-27a 37. These miRNAs were also included in our prediction of the 4 candidate circRNAs. For example, rno_circ_0045161 was the sponge of miR-10b-3p and miR-23a-5p and rno_circ_0028462 was the sponge of miR-27a-3p. MiR-10b-3p was downregulated during focal cerebral ischemia and could alleviate I/R injury by targeting programmed cell death 5 (PDCD5) 38. MiR-23a-5p is involved in white matter remodeling when facing stroke 39. Besides, miR-23a-5p was up-regulated and exacerbated intestinal I/R injury by promoting oxidative stress via targeting peroxisome proliferators activated receptor alpha (PPARα) 40. MiR-27a-3p takes part in the process against apoptosis and oxidative stress to alleviate brain I/R injury symptoms 41. Additionally, miR-27-3p could induce expression of claudin-5 and occludin by downregulating glycogen synthase kinase 3 beta (GSK3ß) and activating the Wnt/ß-catenin signaling pathway to enhance the BBB maintenance 42. It should be noticed that both rno_circ_0045161 and rno_circ_0037165 could act as a let-7 family sponge. Let-7 family members, which are highly conserved across species in functions and sequence, are closely associated with various brain diseases such as Parkinson's disease and traumatic brain injury 43, 44. They also serve as important modulators to regulate several immune processes, such as promoting the polarization of macrophages into the anti-inflammatory M2 phenotype and directly targeting the cytokines IL-6 and IL-10 45. Besides, let-7 can reduce cell apoptosis, for example by regulating Fas expression and the sensitivity of Fas-mediated apoptosis 46.

To further explore the functions of 4 candidate circRNAs, functional enrichment analysis of the miRNA-targeting mRNAs of every candidate circRNAs were conducted. The KEGG analysis identified many signaling pathways such as TNF, FOXO, Hippo, cGMP-PKG, MAPK, and Wnt signaling pathways, which all have complex interactions. Most of these pathways are involved in the cerebral I/R injury process 47-49. Interestingly, some of these pathways were also obtained in the functional enrichment of all DECs, like cGMP-PKG pathway. It was hypothesized that circRNAs can play the same role in DHCA both in regulating its host gene and acting as ceRNA.

There were still some limitations in our study. Although we selected a total of 30 DECs for RT-qPCR validation, only 4 candidate DECs were verified successfully. Both the designed primers and the low expression level of some circRNAs may contribute to this phenomenon. Besides, due to the limited hippocampus tissue, the downstream miRNAs and miRNA-targeting mRNAs of 4 candidate circRNAs were not selected for further verification and we also failed to explore whether the 4 candidate circRNAs would affect the expression of linear RNAs. It is needed to elucidate the underlying molecular mechanisms between these 4 candidate circRNAs and DHCA in future investigation.

## Conclusion

Our study analyzed the expression profiles of circRNAs in rat hippocampus after DCHA. A total of 35192 circRNAs were identified. Among them, 339 circRNAs were dysregulated, including 194 down-regulated and 145 up-regulated between DHCA and sham groups. After RT-qPCR validation, we confirmed the high expression of rno_circ_0028462 and rno_circ_0037165 and the low expression of rno_circ_0045161 and rno_circ_0019047. Taken together, our results provided a better understanding of circRNAs in DHCA-related cerebral injury.

## Supplementary Material

Supplementary figure and tables.

## Figures and Tables

**Table 1:** The characterizations of DECs selected for RT-qPCR verification.

ID	log2FC	PValue	source_gene_id	source_gene	Chr	start	end	strand	genomic_ length	circRNA_ length	Type
rno_circ_0028462	5.82	3.26E-06	ENSRNOG00000016351	Frrs1	2	220435619	220441299	+	5680	563	exon
rno_circ_0021340	5.69	1.91E-05	ENSRNOG00000020514	Sox6	1	185722676	185727014	+	4338	395	intron
rno_circ_0035220	5.64	5.23E-05	n/a	--	4	132094677	132113888	-	19211	344	intergenic_region
rno_circ_0017358	5.54	9.05E-05	n/a	--	18	48853172	48865729	+	12557	493	intergenic_region
rno_circ_0049747	5.42	6.14E-05	ENSRNOG00000008709	Arhgap32	8	33277812	33296545	+	18733	306	exon
rno_circ_0028047	5.33	1.42E-03	ENSRNOG00000019671	Rsbn1	2	206406385	206407059	+	674	674	exon
rno_circ_0023535	5.24	7.08E-04	ENSRNOG00000014779	Pdcd4	1	274628954	274642449	+	13495	320	exon
rno_circ_0024062	5.20	2.31E-03	ENSRNOG00000018191	Oprm1	1	43507130	43508768	+	1638	874	exon
rno_circ_0037165	5.13	1.45E-03	ENSRNOG00000013072	Plxna4	4	59650881	59651296	-	415	415	intron
rno_circ_0038739	5.13	3.35E-03	ENSRNOG00000060950	--	5	12678554	12682441	-	3887	292	intron
rno_circ_0004551	5.00	1.76E-03	ENSRNOG00000001782	Osbpl11	11	70810416	70811760	-	1344	256	exon
rno_circ_0037247	5.00	5.38E-03	ENSRNOG00000061080	Kmt2c2	4	6219914	6222632	+	2718	246	exon
rno_circ_0027463	4.98	6.43E-03	ENSRNOG00000010695	Pdgfc	2	180036669	180046454	+	9785	255	intron
rno_circ_0002585	4.94	2.38E-03	ENSRNOG00000010257	Cuedc1	10	75508833	75513546	+	4713	380	exon
rno_circ_0030003	4.84	7.73E-03	ENSRNOG00000045639	RGD1560883	2	4434943	4444835	+	9892	319	exon
rno_circ_0024647	-4.45	9.57E-03	ENSRNOG00000031391	Ceacam16	1	80775639	80775966	-	327	327	exon
rno_circ_0054079	-4.48	3.81E-03	ENSRNOG00000018931	Dis3l2	9	94027567	94076328	+	48761	188	exon
rno_circ_0033398	-4.48	7.98E-03	n/a	--	3	45277346	45288132	+	10786	334	intergenic_region
rno_circ_0043052	-4.55	2.41E-03	ENSRNOG00000032391	Esyt2	6	144211591	144219597	+	8006	354	exon
rno_circ_0031837	-4.56	6.18E-03	ENSRNOG00000022162 ENSRNOG00000017583	Pbx3; Mapkap1	3	13496852	13510737	+	13885	486	intron
rno_circ_0045161	-4.63	2.10E-03	ENSRNOG00000005063	Atl1	6	92331696	92349697	+	18001	274	exon
rno_circ_0019047	-4.63	5.37E-03	ENSRNOG00000017724	Sf3b3	19	43394226	43401075	+	6849	241	exon
rno_circ_0035410	-4.69	1.75E-03	ENSRNOG00000006509	Srgap3	4	144656178	144656714	-	536	334	exon
rno_circ_0039004	-4.71	4.72E-03	ENSRNOG00000000145	Pik3r3	5	135105373	135121657	+	16284	235	exon
rno_circ_0033973	-4.80	1.02E-03	ENSRNOG00000012580	Ccdc141	3	63930442	63961037	-	30595	290	exon
rno_circ_0007116	-5.04	9.41E-04	ENSRNOG00000003743	Dars	13	45074545	45086416	-	11871	393	exon
rno_circ_0031737	-5.59	1.93E-05	n/a	--	3	131470577	131475654	+	5077	403	intergenic_region
rno_circ_0030500	-5.59	8.71E-05	n/a	--	2	73750149	73750789	+	640	640	intergenic_region
rno_circ_0004035	-7.89	1.75E-05	ENSRNOG00000030840	Cadm2	11	4324301	4397333	-	73032	441	exon
rno_circ_0016962	-9.72	1.04E-06	ENSRNOG00000012899	Rbbp8	18	3175482	3176344	+	862	862	intron

DECs, differentially expressed circRNAs; RT-qPCR, reverse transcription-quantitative polymerase chain reaction.
