# Supplementary material for: Transcriptomic Profiling of circRNAs in rat Hippocampus after Deep Hypothermic Circulatory Arrest
Source: Int J Med Sci. 2023 Apr 1;20(5):627–38. doi: 10.7150/ijms.82503 (PMC10110479; doi:10.7150/ijms.82503)
Supplement: Supplementary file 1 — Supplementary figure and tables. [file ijmsv20p0627s1.pdf]

Transcriptomic Profiling of circRNAs in rat Hippocampus after Deep Hypothermic Circulatory Arrest

Tianlong Wang [1 #], Weidong Yan [1 #], Shengqiang Pei [2], Mingru Zhang [3], Qiaoni Zhang [1], Yuan Teng [1], Gang Liu [1], Jian Wang [1], Shujie Yan [1], Bingyang Ji [1 *]

[1.] Department of Cardiopulmonary Bypass, Fuwai Hospital, National Center for Cardiovascular Disease, State Key Laboratory of Cardiovascular Medicine, Chinese Academy of Medical Sciences & Peking Union Medical College, Beijing 100037, China

[2.] State Key Laboratory of Cardiovascular Disease, Beijing Key Laboratory for Molecular Diagnostics of Cardiovascular Diseases, Diagnostic Laboratory Service, Fuwai Hospital, National Center for Cardiovascular Diseases, Chinese Academy of Medical Sciences & Peking Union Medical College, Beijing, China

[3.] Department of Anaesthesiology, Beijing Tongren Hospital, Capital Medical University, Beijing, China

[#] These authors contributed to this work equally.

[*] Corresponding author: Bingyang Ji, MD, PhD, Department of Cardiopulmonary Bypass, National Center for Cardiovascular Disease and Fuwai Hospital, No. 167 Beilishi Road, Xicheng District, 10010, Beijing, China.

Phone: +86-10-88398285. Email: jibingyang@fuwai.com

Supplementary Figure

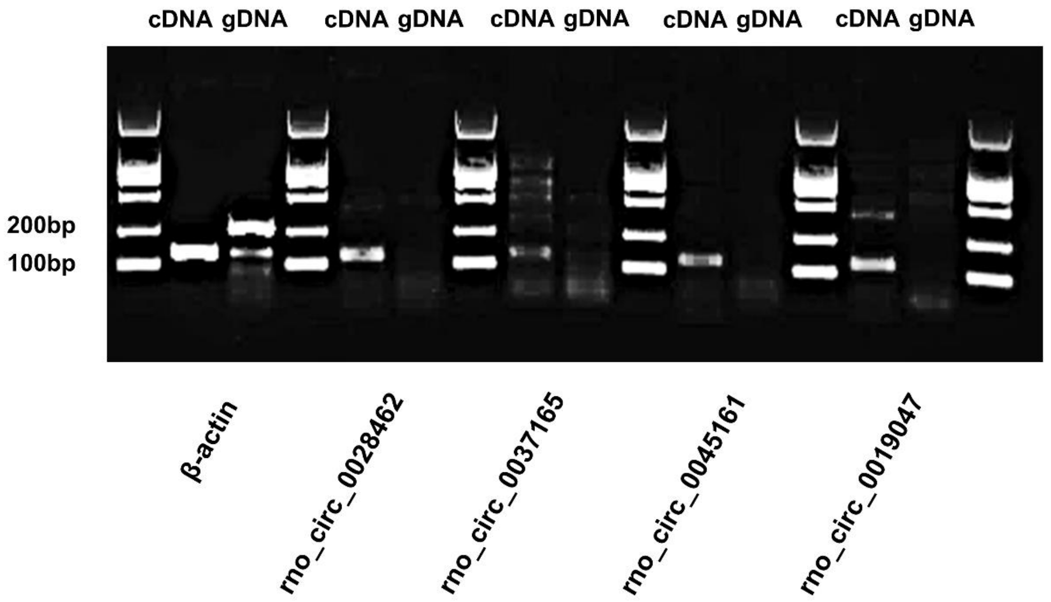

Figure S1. The existence of 4 candidate circRNAs were validated by agarose gel electrophoresis.

cDNA: complementary DNA; gDNA: genomic DNA.

Supplementary Tables

Table S1. The sequences of primers for reverse transcription-quantitative polymerase chain reaction.

**Primer**	**Forward**	**Reverse**
rno_circ_0028462	GAAATGAGCGGACCCAGTGA	CGCTCAAATCCTCAGCGTTC
rno_circ_0021340	AGGTCTAGGAAGCTCACTCCA	GCACCCAGAAGTCACTGGTA
rno_circ_0035220	AAGATAGATCACTTCCTGGTGCC	GCATAGCACACAATGCAGGT
rno_circ_0017358	TTGGAGAATTACGATTAGGAGCCC	TCTCGGTGATATAAGCTGGTGC
rno_circ_0049747	AAGAGCTGGTCTACCTCGTG	CATGCTTGACCTTCATGCTCG
rno_circ_0028047	CCCAACACTCCAGTGAAAATGG	ACAAAATCATCAGCTCTCTGGGT
rno_circ_0023535	GCTACTGTGCTCCTGAGTATGT	TTCACTTGCACTGATTATACGCAA
rno_circ_0024062	CCACTCGAGTCCGTCAGAAC	AAAGGGCAGTGTACTGGTCG
rno_circ_0037165	TCCATGGGAACGAGCTTTAC	TTCCTCCCTTCCTTCCTTTC
rno_circ_0038739	AAGAAGCTTTTGGTCTTCGGGA	TGATGACTCAAGCCTTCCATC
rno_circ_0004551	GTACACAGCACCACACGGAA	CAAGCTGCAAAGTCCCTCTG
rno_circ_0037247	GGACAGGACAAGGAAAAGCC	TCCCGCTCAGGACTAGATTCA
rno_circ_0027463	GCGGATCCAGCTGACATTTG	CTCTCATGCCGGGGATCTTG
rno_circ_0002585	GGCCATGCTTATGAGGAAGACT	AACGGCCACATTGCTGGATT
rno_circ_0030003	GACTGGTCAAACTTGCTGCC	CCCAGTTTCTCCTTGCTCCA
rno_circ_0005842	AAAACCAACCAGGGAGTGGG	TTCTCGACCTCCTCTTCGGT
rno_circ_0054079	CCATGACTGACAAGCTGACCT	GAAGCATGGGGACCACCGT
rno_circ_0033398	GAGTCAAGGAAGCCAGGATGAT	CCACCAGTGAGGCCTGTTCA
rno_circ_0043052	AAATCAGACCCCTATGGAATCATCC	TGTCCAGTTAATCTCTAAAAGCTCA
rno_circ_0031837	CTCGAGCTGGGACTTTGGTAT	GAGCTTGCCCAGGGAGTATTT
rno_circ_0045161	TCTCCTACCTCATCCTGGCTT	TCGTTCAGATCCACCTCTCCA
rno_circ_0019047	CTGTTGCTGCTGCTATGTGTG	CTGTGACACTCAGTCACGTCT
rno_circ_0035410	CACCATGAAGCCATCTTCCCC	ATGAGGATCTGCTGGATTGGG
rno_circ_0039004	ACCTGCAAGAATTCCACTCTCA	TGCTGACGTCACTGGCTTAG
rno_circ_0033973	TTAAAGACTCTACAGCCGCCG	GAACCCAGACTCTGTTCCTGC
rno_circ_0007116	TCAATGTTCCCTCGTGACCC	AAATGCCGGACTGGAGATGG
rno_circ_0031737	ACAGTAGGTGGAAGCAACGG	CTGGTGTGTGCCCTCCATTA
rno_circ_0030500	ATATCCCCTTGGTCCCTTCA	GCTGAAGATTTCGCCATACC
rno_circ_0004035	AAGACGTCAGGCAGTAAGCC	TCAGAATTGCAGTTCCGCCT
rno_circ_0016962	TTTGCAGGTTTACAAATCAAAG	GTCTTTGGAAAACAACTTAGCAA
β-Actin	GGAGATTACTGCCCTGGCTCCTA	GACTCATCGTACTCCTGCTTGCTG

Table S2. Integrated genome viewer (IGV) screen shots of 4 validated circRNAs.

<table>
<tr><td colspan="2">rno_circ_0028462</td></tr>
<tr><td>Chr location</td><td>chr 2: 220435619-220441299
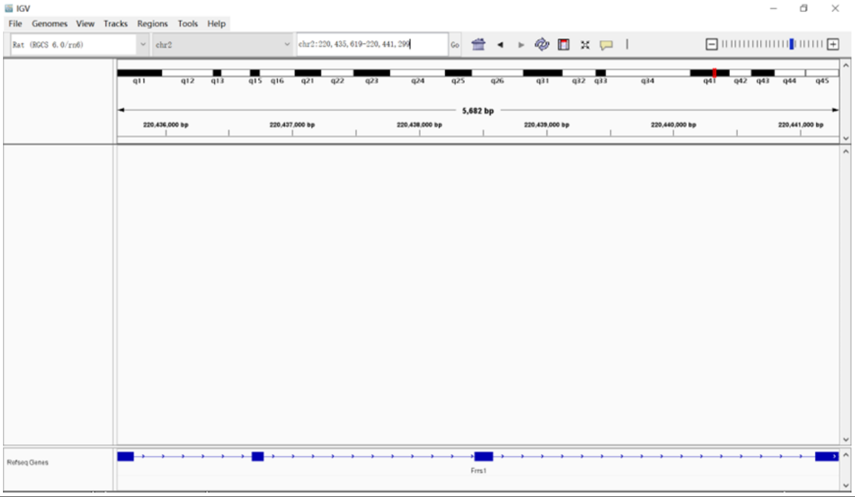

</td></tr>
<tr><td>circRNA feature</td><td>exon:220435620-220435756;
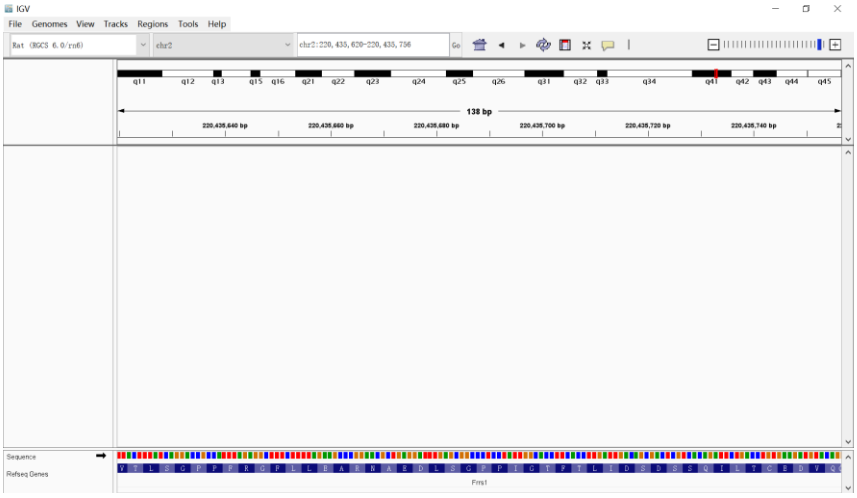

exon:220436683-220436777;
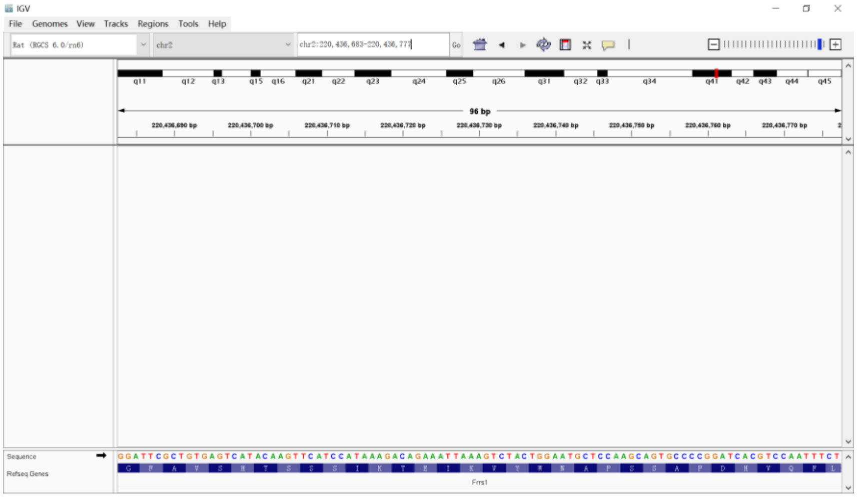

exon:220438434-220438581;</td></tr>
</table>

	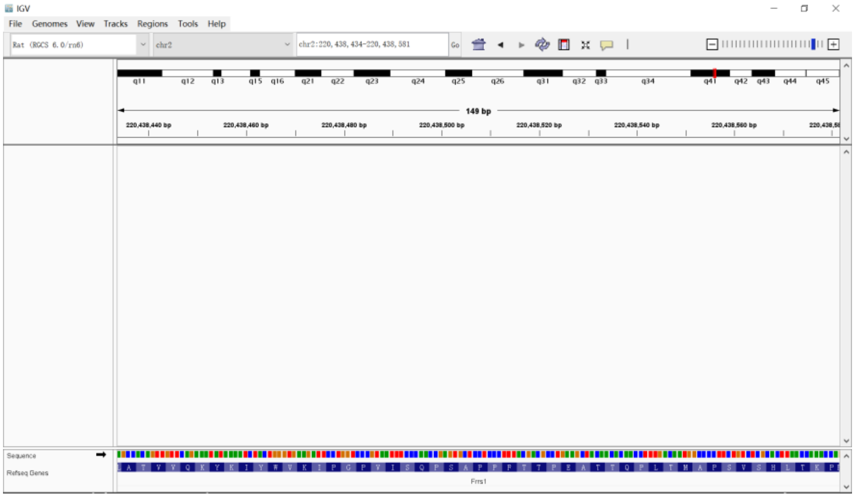<br><br>exon:220441117-220441299;<br>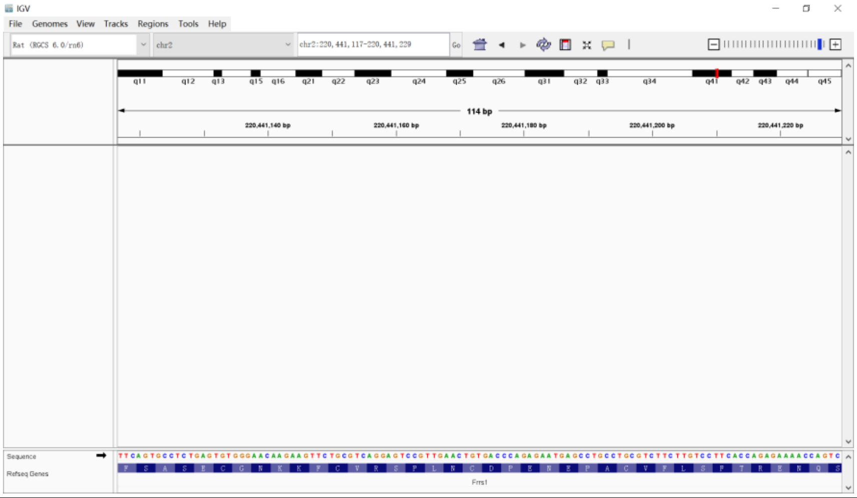
**rno_circ_0037165**	
Chr location	chr 4: 59650881-59651296<br>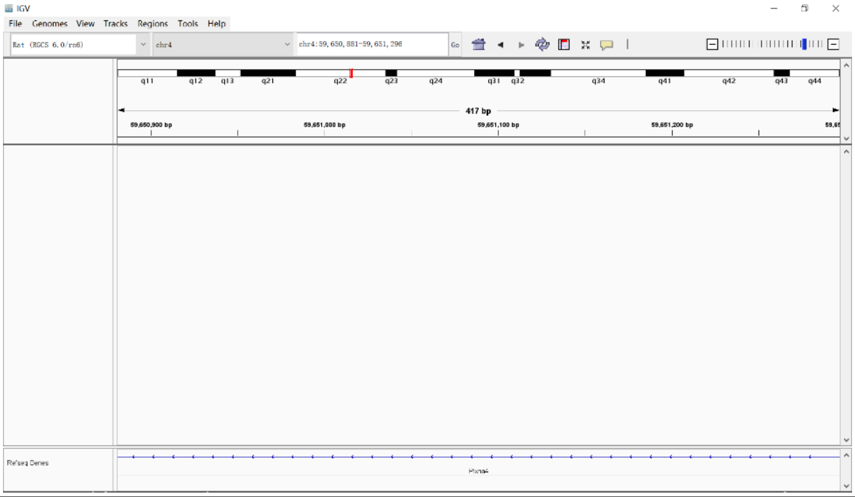
circRNA feature	intron:59650881-59651296;

	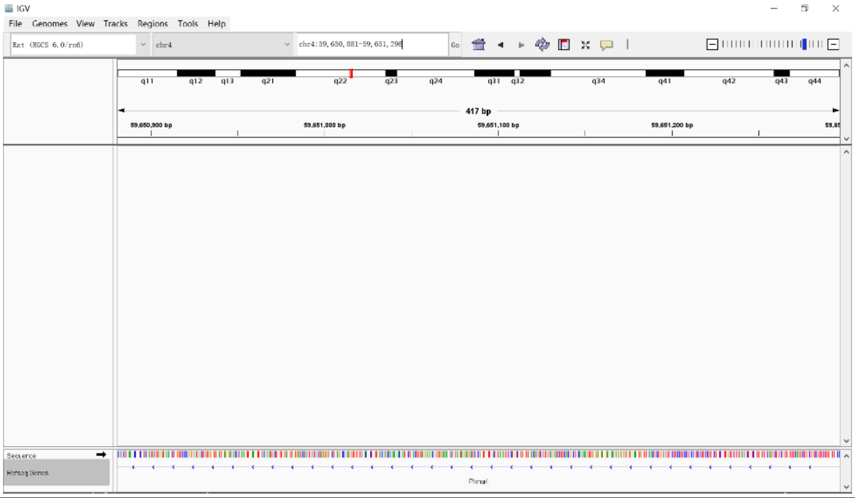
**rno_circ_0045161**	
Chr location	chr 6: 92331696-92349697 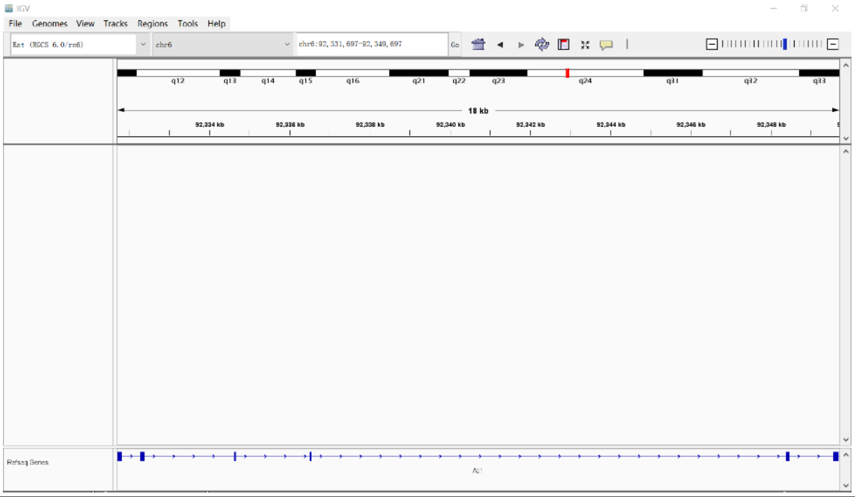
circRNA feature	exon:92331697-92331831; 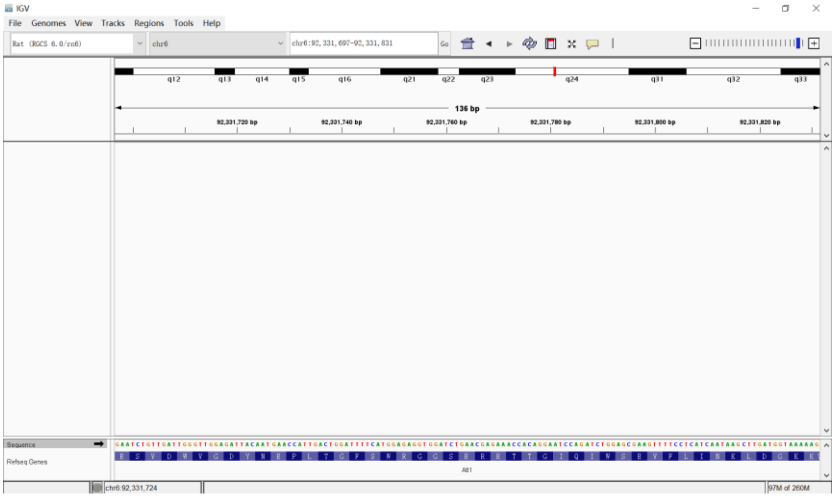

	exon:92349559-92349697;<br>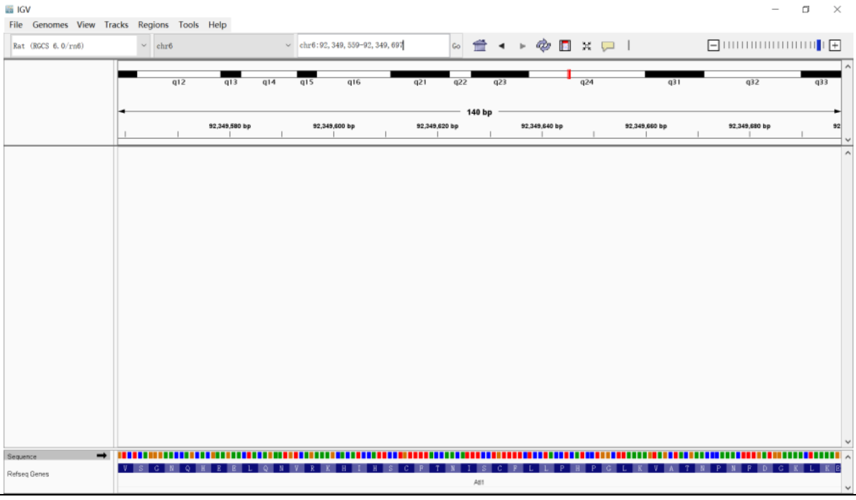
**rno_circ_0019047**	
Chr location	chr 19: 43394226-43401075<br>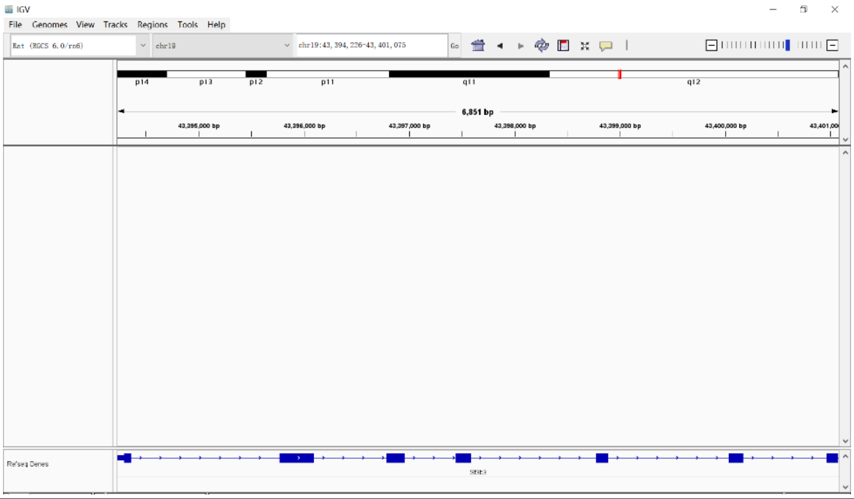
circRNA feature	exon:43394227-43394363;<br>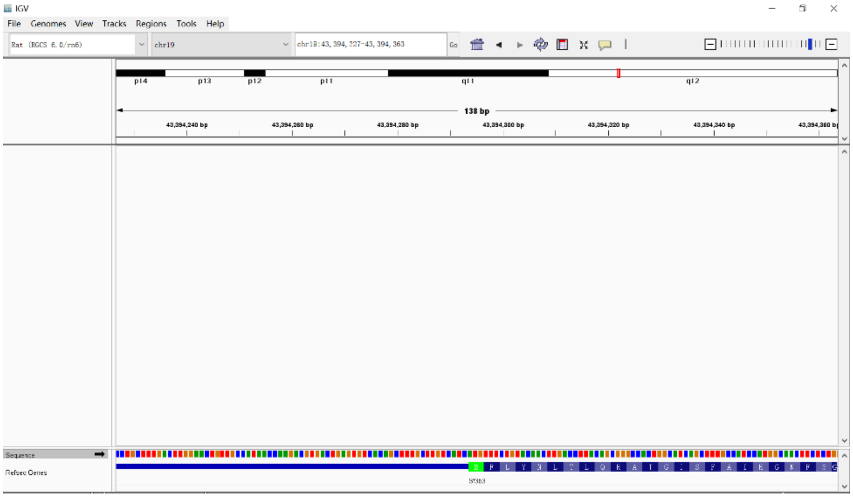<br>exon:43400972-43401075;

	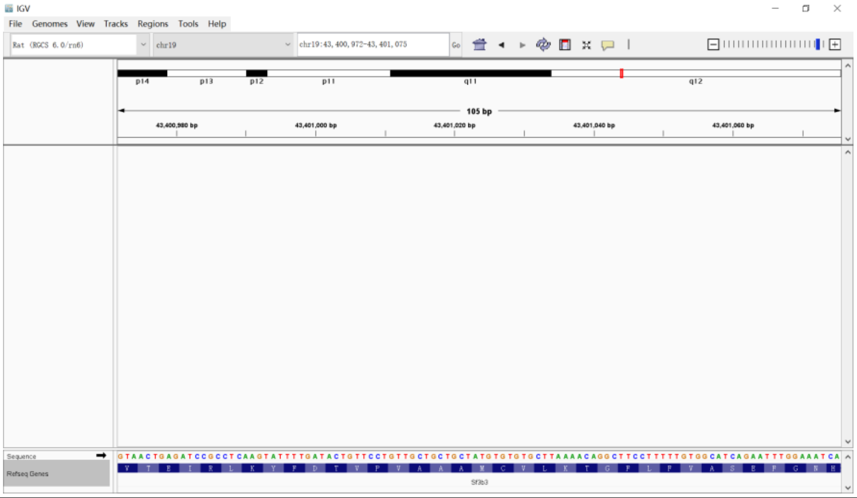

IGV
File Genomes View Tracks Regions Tools Help
Rat (RGCS 6.0/rn6)
chr19
chr19:43,400,972-43,401,075
Go
p14
p13
p12
p11
q11
q12
105 bp
43,400,980 bp
43,401,000 bp
43,401,020 bp
43,401,040 bp
43,401,060 bp
Sequence
Refseq Genes
Sf3b3
